# Jia-Wei-Jiao-Tai-Wan ameliorates type 2 diabetes by improving β cell function and reducing insulin resistance in diabetic rats

**DOI:** 10.1186/s12906-017-2016-5

**Published:** 2017-11-29

**Authors:** Guang Chen, Xueping Yang, Xiaoyu Yang, Lingli Li, Jinlong Luo, Hui Dong, Lijun Xu, Ping Yi, Kaifu Wang, Xin Zou, Fuer Lu

**Affiliations:** 1 Department of Integrative Traditional & Western Medicine, Tongji Hospital, Tongji Medical College, Huazhong University of Science & Technology, Wuhan, 430030 China; 2 Institute of Integrative Traditional & Western Medicine, Tongji Hospital, Tongji Medical College, Huazhong University of Science & Technology, Wuhan, 430030 China; 3 Department of Oncology, Xiangyang No. 1 Hospital, Xiangyang, 441000 China; 4 Department of Traditional Chinese Medicine, Pu’ai Hospital, Tongji Medical College, Huazhong University of Science & Technology, Wuhan, 430033 China; 5 Department of Emergency, Tongji Hospital, Tongji Medical College, Huazhong University of Science & Technology, Wuhan, 430030 China

**Keywords:** Jia-Wei-Jiao-Tai-Wan (JWJTW), Type 2 diabetes mellitus, Pancreatic β cell, Oxidative stress, Insulin resistance

## Abstract

**Background:**

Jia-Wei-Jiao-Tai-Wan (JWJTW), composed of Jiao-Tai-Wan (*Cinnamomum cassia* and *Rhizoma coptidis*) and other antidiabetic herbs, including *Astragalus membranaceus*, *Herba Gynostemmatis*, *Radix Puerariae Lobatae*, *Folium Mori* and *Semen Trigonellae*, is widely used to treat diabetes and has demonstrated a curative effect in the clinic, but the potential mechanism is unknown. This study aimed to explore the effects of JWJTW on diabetic rats and to clarify the underlying mechanism.

**Methods:**

JWJTW was prepared, and the main components contained in the formula were identified by high-performance liquid chromatography (HPLC) fingerprint analysis. Diabetic rats induced by streptozotocin (STZ) and a high-sucrose-high-fat diet were treated with two concentrations of JWJTW (1.025 and 2.05 g/kg/d) for 100 days. The oral glucose tolerance test (OGTT), insulin release test (IRT) and insulin tolerance test (ITT) were performed to measure the glycometabolism of the diabetic rats at the end of the treatment period. Blood was collected to determine the serum lipid levels of the diabetic rats. Nitric oxide (NO), malondialdehyde (MDA), superoxide dismutase (SOD) and glutathione peroxidase (GSH-px) were detected in pancreas homogenates to analyze the oxidative stress in the pancreata of diabetic rats, and the expression levels of pancreatic and duodenal homeobox 1 (PDX-1) and insulin in the pancreas were tested by Western blot to measure pancreatic islet function. In addition, Western blots were used to measure the expression of proteins related to the insulin signaling pathway in skeletal muscle of the diabetic rats.

**Results:**

The results showed that the administration of JWJTW could ameliorate impairments in glucose tolerance, insulin release function and insulin tolerance in diabetic rats. JWJTW could also dose-dependently reduce serum lipid levels in diabetic rats. JWJTW restrained oxidative stress by decreasing the expression of NO and MDA and increasing the expression of SOD and GSH-px. JWJTW improved the function of pancreatic β cells by increasing PDX-1 and insulin expression. In addition, JWJTW restored the impaired insulin signaling; upregulated phospho-insulin receptor (pInsR) expression, insulin receptor substrate (IRS) tyrosine phosphorylation, phosphatidylinositol 3-kinase (PI3K) (p85), and glucose transporter 4 (GLUT4) expression; and downregulated the serine phosphorylation of IRS.

**Conclusions:**

This study suggests that JWJTW can ameliorate type 2 diabetes by improving β cell function and reducing insulin resistance in diabetic rats.

## Background

Diabetes mellitus is a continuously growing health problem that causes substantial morbidity, mortality and long-term complications. In 2015, the International Diabetes Federation estimated that, 415 million adults aged 20–70 years worldwide had type 2 diabetes mellitus (T2DM); 75% of those affected lived in low-and middle-income countries, 46.5% had not been diagnosed and approximately 5 million people died of diabetes each year. By 2040, 642 million people are expected to suffer from diabetes [1]. Diabetes consumes approximately 12% of the world’s medical costs (approximately $673 billion), so there is a worldwide search for better pharmacologic agents to control T2DM.

T2DM is characterized by the dysregulation of carbohydrate, lipid and protein metabolism and it results from impaired insulin secretion, insulin resistance (IR) or a combination of both [2]. IR refers to a reduction in the efficiency by which insulin promotes glucose uptake and utilization by peripheral tissues, such as liver, muscle and adipose tissue. Under these circumstances, excessive insulin is secreted by body to maintain the stability of blood glucose, leading to hyperinsulinemia; thus, it is important to repair the insulin signaling pathway of peripheral tissue in the treatment of T2DM. Impaired insulin secretion is caused by the apoptosis of pancreatic β cells, resulting in insufficient insulin production that can no longer meet physiological requirements. Thus, the achievement of durable glycemic control requires antidiabetic medications that can reverse the pathophysiological defects present in T2DM [3, 4], such as the apoptosis of pancreatic β cells and peripheral tissue IR.

Diabetes has a long history in ancient China and other countries and more and more increasing numbers of herbs [5] have been widely used as medicines in the treatment of T2DM. Jiao-Tai-Wan, consisting of *Rhizoma Coptidis* and *Cortex Cinnamomi cassiae*, was first described by Han Yi (in the Chinese Ming Dynasty) in his treatise “Han Si Yi Tong”. Jiang et al. demonstrated that Jiao-Tai-Wan could decrease blood lipid levels and increase insulin sensitivity in diabetic rats [6]. Zou et al. revealed the effects of Jiao-Tai-Wan on the deposition of fat in the pancreas and the apoptosis of pancreatic islet β cells [7]. Our previous research also indicated an antihyperglycemic effect of Jiao-Tai-Wan in diabetic rats [8]. However, because the combination of two herbs is too simple to treat the complex symptoms of diabetic patients, the clinical application of Jiao-Tai-Wan has been limited. Jiao-Tai-Wan was improved by the addition of other antidiabetic herbs, including *Astragalus membranaceus*, *Herba Gynostemmatis*, *Radix Puerariae Lobatae*, *Folium Mori* and *Semen Trigonellae*, to form Jiao-Wei-Jiao-Tai-Wan (JWJTW). Sun et al. demonstrated that *Astragalus membranaceus* could inhibit the extrinsic and intrinsic apoptotic pathways in high-glucose-stimulated H9C2 cells [9]. Gypenosides extracted from *Herba Gynostemmatis* also had a lipid-lowering effect on nonalcoholic fatty liver disease (NAFLD) rats [10]. Researchers have revealed that puerarin, which is the main component of *Radix Puerariae Lobatae*, attenuates IR in 3T3L1 cells and diabetic rats [11–13]. Moreover, *Folium Mori* [14, 15] and *Semen Trigonellae* [16] also have antidiabetic effects on diabetic rats. JWJTW also had a great hypoglycemic effect in clinical T2DM treatment, and a patent has been issued (IPC Classification Nos. A61K36/185, A61P3/10, A61P3/06, A61P3/00 and A61K36/718). It was demonstrated that JWJTW could ameliorate oxidative stress and reduce the apoptosis of retinal ganglion cells in diabetic rats [17]. In addition, JWJTW was reported to reduce the fasting blood glucose (FBG) levels of T2DM patients in a clinical trial [18]. On the basis of these research studies and clinical applications of JWJTW to treat T2DM, a further animal experiment was conducted by us to analyze the potential mechanism of the antidiabetic action of JWJTW.

## Methods

### Herbal materials and the preparation of JWJTW

**Fig. 1:**
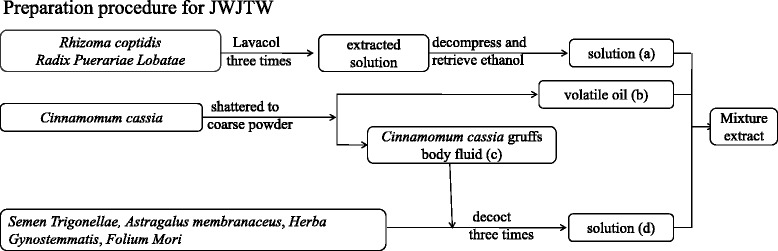
Preparation procedure for JWJTW

The medicinal plants used to prepare JWJTW are listed in Table 1. These plants were purchased from Hubei Herbal Materials Company (Wuhan, China). The voucher specimens were conserved at the herbal herbarium of Hubei University of Traditional Chinese Medicine, and were authenticated by Prof. Keli Chen at the School of Pharmacy, Hubei University of Chinese Medicine, based on their microscopic and macroscopic characteristics. JWJTW decoction was prepared in the Institute of Integrative Traditional Chinese and Western Medicine, Tongji Hospital, Tongji Medical College, Huazhong University of Science & Technology (HUST, China). The preparation procedure (Fig. 1) was as follows: *Rhizoma coptidis* and *Radix Puerariae Lobatae* were extracted three times with Lavacol, and the extracting solutions were combined. The ethanol in the combined extracting solution was decompressed and retrieved as solution (a) with no ethanol flavor. *Cinnamomum cassia* was shattered to a coarse powder and then extracted as a volatile oil (b) with wet distillation. The *Cinnamomum cassia* gruff body fluid was reserved as (c). *Semen Trigonellae, Astragalus membranaceus*, *Herba Gynostemmatis*, *Folium Mori* and (c) were decocted three times after the addition of distilled water. The above solution (d) was concentrated with decompression. The concentrated extraction was added to extracts (a) and (b). The mixture was stored at 4 °C after vacuum dehydration.

**Table 1:** Composition of Jia-Wei-Jiao-Tai-Wan (JWJTW)

Medicinal plant	Botanical plant name	Amount (g)
*Rhizoma coptidis*	*Coptis chinensis* Franch	3.0
*Cinnamomum cassia*	*Cinnamomum cassia* Presl.	1.5
*Radix Astragalus*	*Astragalus membranaceus* (Fisch.) Bge. var. *monholicus* (Bge.) Hsiao	7.5
*Herba Gynostemmatis*	*Gynostemma pentaphyllum* (Thunb.) Makino	9.0
*Radix Puerariae Lobatae*	*Pueraria lobata* (Willd.) Ohwi	7.5
*Folium Mori*	*Morus alba* L.	6.0
*Semen Trigonellae*	*Trigonella foenum-graecum* L.	6.0

### High-performance liquid chromatography (HPLC) fingerprint of the extract

HPLC fingerprinting of the JWJTW extract was performed to identify the main chemical constituents in the samples. Trigonelline, puerarin, coptisine, jateorhizine, berberine, palmatine and cinnamic acid were used as standard substances. The extract was dissolved in water at a concentration of 1 g/ml (*w*/*v*) and then was diluted with methanol–water (50:50) to 0.5 g/ml (w/v). HPLC analysis was performed using an Eclipse XDB-C18 column (4.6 × 250 mm, 5 μm, Aglient, USA) eluted with mobile phases of methanol (A) and 0.1% phosphoric acid (B) with gradient elution (Table 2). The flow rate was 1.0 ml/min and the detection wavelength was 280 nm.

**Table 2:** The mobile phases of HPLC fingerprint

Time (min)	Methanol (A)	0.1% phosphoric acid (B)
0	5	95
10	5	95
110	55	45
113	5	95
120	5	95

### Animals and treatment

Healthy male Wistar rats aged 9 weeks (220-250 g) were purchased from Hubei Province Experimental Animal Research Center (grade specific pathogen-free). The animals were housed in an environmentally controlled breeding room (temperature: 20 ± 2 °C, humidity: 60 ± 5%, 12-h dark/light cycle). Water and food were given ad libitum. The animal studies were overseen and approved by the Animal Ethics Committee of Tongji Medical College, HUST before and during the experiment ([2015] IACUC Number: 305). After one-week of adaptive feeding, the rats were fasted for 12 h and were injected with streptozotocin (STZ, Sigma Chemical Company, St. Louis, Missouri) at a dose of 30 mg/kg through the tail vein. STZ was dissolved in sodium citrate saline buffer (pH 4.5) and injected immediately within a few minutes to avoid degradation. Then, the rats were fed a high-sucrose-high-fat diet. Two weeks after the injection, impaired glucose tolerance (IGT) rats, as determined by an oral glucose tolerance test (OGTT), were randomly divided into three groups as follows: (1) 1.025 g/kg/d JWJTW group (*n* = 11), intragastric administration (ig) of 1.025 g/kg/d JWJTW; (2) 2.05 g/kg/d JWJTW group (*n* = 11), ig 2.05 g/kg/d JWJTW; and (3) diabetic group (*n* = 11), ig the vehicle for JWJTW. Another 11 rats established as a normal group received neither STZ injection nor drug administration. The high-sucrose-high-fat diet (sucrose: lard: milk powder: egg: conventional feed = 30:40:8:4:63) was given to all the rats except for the normal group during the entire treatment period. Body weight was measured weekly to adjust the dose of JWJTW.

### Oral glucose tolerance test (OGTT), insulin release test (IRT) and insulin tolerance test (ITT)

OGTT was performed 3 months after the first experimental treatment. Having been fasted for 12 h, the rats were orally administered 2.2 g/kg glucose. Then, blood samples were collected from the tail vein at 0 (just before the glucose load), 30, 60, 120 and 180 min (after the glucose load) for glucose and insulin assays. ITT was performed 1 week after OGTT. Baseline glucose levels were determined after a 4 h fast. Insulin (0.4 units/kg) was injected subcutaneously. Then, blood samples were collected from the tail vein at 0 (just before the insulin injection), 30, 60, 90 and 120 min (after the insulin injection) for the glucose assay.

### Tissue preparation

The anesthetic drug sodium pentobarbital (80 mg/kg, Goodbio, Hubei, China) and all other necessary measures were used to reduce animal suffering during experimental procedures. After anesthesia, blood samples were collected from the internal canthus vein and sera were prepared and stored at −80 °C for the determination of serum lipid levels. To assess the effect of JWJTW on insulin receptor activation in vivo, rats were anesthetized with sodium pentobarbital at the end of the 100-day treatment period. Then, insulin (8 units/kg weight) or saline was injected into the vein of rats, as previously described [19]. Rats were sacrificed with carbon dioxide euthanasia approximately 4 min after the insulin injection. After the animal’s death was confirmed the soleus muscles were separated, washed with cold phosphate buffer, and cut into 200–300 mg portions, which were then immediately frozen in liquid nitrogen and stored separately at −80 °C. The pancreas was cut in half after dissection, with one part immediately frozen in liquid nitrogen and stored at −80 °C and the other fixed in 4% paraformaldehyde.

### Measurement of glucose and lipid metabolic parameters

Blood glucose was estimated with a commercially available glucose kit (Beijing North Kangtai Clinical Reagent Co., Ltd., China) based on the glucose oxidase method. Lipid parameters such as total cholesterol (TC), triglyceride (TG), low-densitylipoprotein (LDL) and free fatty acids (FFA) were measured following the manufacturer’s instructions (Wenzhou Dongou Biological Engineering Co., Ltd., China).

### Nitric oxide (NO), malondialdehyde (MDA), superoxide dismutase (SOD) and glutathione peroxidase (GSH-px) levels in pancreas homogenates

The contents of NO, MDA, SOD and GSH-px in the pancreas were measured as follows. The pancreas was thawed, weighed and homogenized with 0.9% saline. Then, the homogenate was centrifuged (12,000 *g*, 15 min, 4 °C) and the supernatant was immediately used for the assessment of NO, MDA, GSH-px and SOD. The NO, MDA, SOD and GSH-px levels were measured with commercial kits (Nanjing Jiancheng Biological Engineering Institute).

### Histological staining

The general morphology of formalin-fixed paraffin-embedded pancreatic tissue sections (4 μm) was determined by hematoxylin and eosin staining.

### Antibodies and chemicals

The antibodies used in this study were purchased from the following manufacturers: the antibody against pancreatic and duodenal homeobox 1 (PDX-1) was purchased from Chemi-con (USA); against insulin and against insulin receptor (InsR-β) from Abcam (UK); against insulin receptor substrate (IRS)-1 from Upstate (USA); against phospho-insulin receptor substrate (pIRS)-1(Ser307) and pIRS-1(Tyr612), phospho-insulin receptor (pInsR)-β (Tyr1361), phosphatidylinositol 3-kinase (PI3K) (p85) and glucose transporter 4 (GLUT4) from Cell Signaling Technology (CST, USA) and against GAPDH from Wuhan Gugeshengwu Technology Co., Ltd. (China). SDS-PAGE gel preparation, phenylmethanesulfonyl fluoride (PMSF), protease inhibitor cocktail, eosin, and hematoxylin were purchased from Wuhan Goodbio Technology Co., Ltd. (China).

### Western blot analysis

The expression of proteins involved in the insulin signaling pathway in skeletal muscle and the expression of PDX-1 and insulin in the pancreas were measured by Western blot. The proteins were separated via 10–12% SDS-PAGE (80 V, 0.5 h and then 120 V, 1 h) and transferred to a 0.22 μm nitrocellulose membrane (280 A, 1 kDa/min). The membranes were blocked with bovine serum albumin (BSA) powder in ultrapure water for 1 h at room temperature and incubated with primary antibodies overnight at 4 °C. The next day, secondary antibodies were applied to the membranes and incubated for 1 h at room temperature following 3 rounds of interval washes with Tris-buffered-saline and Tween 20 (TBST, 10 min each). Bands were visualized with Odyssey Infrared Imaging (LI-COR Biosciences, USA) and were normalized against GAPDH using Image-Pro Plus software (Media Cybernetics, USA).

### Statistical analysis

Statistical analyses were performed using SPSS 19.0 software (SPSS Software Products, C hicago, IL, USA). All results are expressed as the means ± S.D. Significant differences among the groups were evaluated with a one-way analysis of variance (ANOVA) and Dunnett’s t-test, and *p* < 0.05 was considered significant.

## Results

### HPLC fingerprint of the extract

**Fig. 2:**
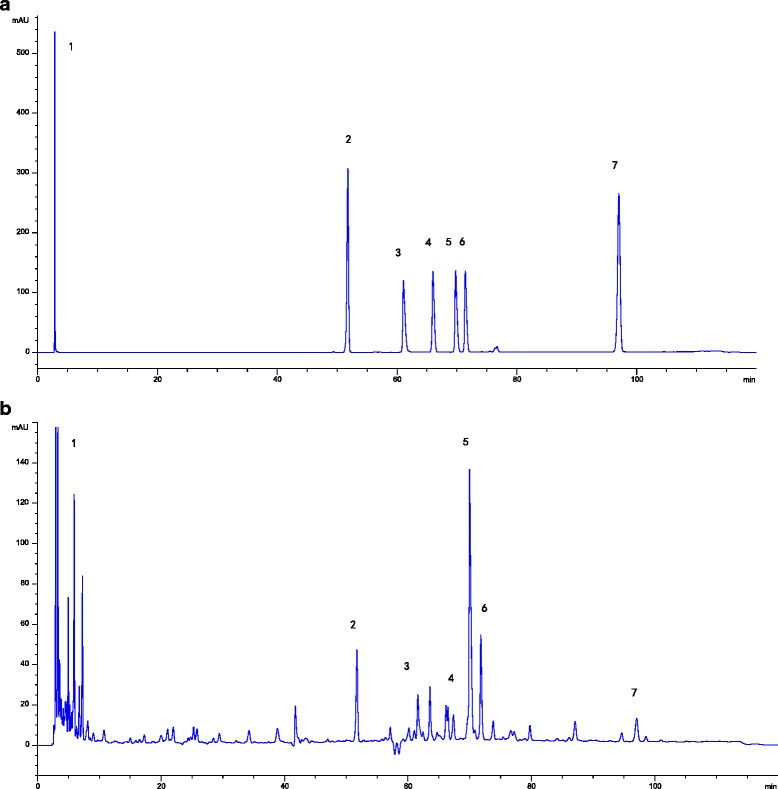
HPLC fingerprint of the extract. HPLC fingerprint chromatograms of extracts of the reference standards (**a**) and JWJTW (**b**). In the chromatograms: (1) trigonelline (PubChem CID:5570); (2) puerarin (PubChem CID: 5,281,807); (3) coptisine (PubChem CID: 72,322); (4) jateorhizine (PubChem CID:72,323); (5) berberine (PubChem CID: 2353); (6) palmatine (PubChem CID: 19,009); (7) cinnamic acid (PubChem CID:444,539)

HPLC fingerprint chromatograms of JWJTW are shown in Fig. 2. By comparison with both the retention times and UV spectra of the reference standards, seven compounds in the extract were well identified. The compounds were as follows: trigonelline (PubChem CID: 5570), puerarin (PubChem CID: 5,281,807), coptisine (PubChem CID: 72,322), jateorhizine (PubChem CID:72,323), berberine (PubChem CID: 2353), palmatine (PubChem CID: 19,009) and cinnamic acid (PubChem CID:444,539).

### Effects of JWJTW on glucose tolerance, insulin release and insulin tolerance in diabetic rats

**Fig. 3:**
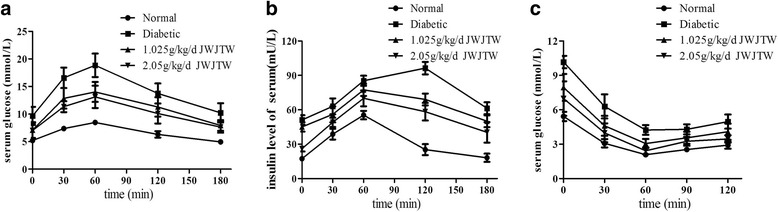
Effects of JWJTW on glucose tolerance, insulin release and insulin tolerance in diabetic rats. **a** GTT of diabetic rats; **b** IRT of diabetic rats; **c** ITT of diabetic rats. Normal: normal group rats without streptozotocin (STZ) or high-sucrose-high-fat diet; Diabetic: diabetic rats induced by STZ and a high-sucrose-high-fat diet; 1.025 g/kg/d JWJTW: diabetic rats treated with 1.025 g/kg/d Jia-Wei-Jiao-Tai-Wan; 2.05 g/kg/d JWJTW: diabetic rats treated with 2.05 g/kg/d Jia-Wei-Jiao-Tai-Wan. GTT and IRT: Oral glucose tolerance test and insulin release test, respectively, performed on diabetic rats that were orally administered 2.2 g of glucose per kg after 12 h of fasting (*n* = 10). ITT: insulin tolerance test performed on diabetic rats injected with 0.4 U insulin per kg after 4 h of fasting (*n* = 10)

After 100 days of treatment with JWJTW, the serum glucose levels at 0, 30, 60, 120 and 180 min in the diabetic group were higher than those in the normal group. The serum glucose levels at all of the above time points were lower in the JWJTW group than in the control group (Fig. 3a). As shown in Fig. 3b, diabetic group mice had higher serum insulin levels, but the release of insulin was delayed compared with the normal group; however, this was reversed with JWJTW treatment. The results of the ITT showed that significant IR had developed in the diabetic rats. Treatment with JWJTW dramatically decreased glucose levels at each point, indicating that insulin sensitivity had improved (Fig. 3c).

### Effects of JWJTW on lipid metabolic parameters of diabetic rats

**Fig. 4:**
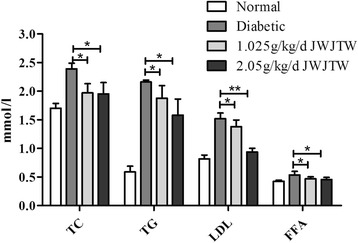
Effects of JWJTW on lipid metabolic parameters of diabetic rats. TC: total cholesterol, TG: triglyceride, LDL: low-density lipoprotein, FFA: free fatty acids. ^*^ *p* < 0.05, ^**^ *p <* 0.01 (*n* = 10)

In rats administered STZ and then fed a high-sucrose-high-fat diet for 100 days, TC,TG, LDL and FFA were all significantly elevated (Fig. 4), indicating that hyperlipidemia symptoms were also successfully established in the diabetic group. Daily treatment with JWJTW for 100 days dramatically decreased the TC, TG, LDL and FFA levels in serum (^*^ *p* < 0.05, ^**^ *p* < 0.01).

### Effects of JWJTW on the expression of oxidative stress in pancreas homogenates of diabetic rats

**Fig. 5:**
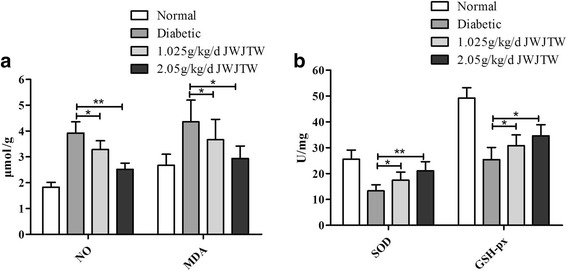
Effects of JWJTW on SOD and GSH-px activity, MDA and NO content of pancreatic islets in diabetic rats. NO: nitric oxide, SOD: superoxide dismutase, GSH-px: glutathione peroxidase, MDA: malondialdehyde. **a** Homogenates of the pancreas were analyzed for NO and MDA. **b** Homogenates of the pancreas were analyzed for SOD and GSH-px. ^*^ *p* < 0.05, ^**^ *p <* 0.01 (*n* = 10)

MDA, an important index for judging lipid peroxidation, was significantly increased in diabetic rats. After the treatment with JWJTW, the content of MDA in the pancreas was markedly decreased (^*^ *p* < 0.05). Similar results were observed NO, a type of reactive nitrogen species that can activate the oxidative stress mechanism (Fig. 5a, ^*^ *p* < 0.05, ^**^ *p* < 0.01). As shown in Fig. 5b, compared with the normal group, there were significant decreases in SOD and GSH-px in the diabetic group, and the trend was reversed in diabetic rats treated with JWJTW (^*^ *p* < 0.05, ^**^ *p* < 0.01).

### Effects of JWJTW on pancreatic islet number and structure of islets in diabetic rats

**Fig. 6:**
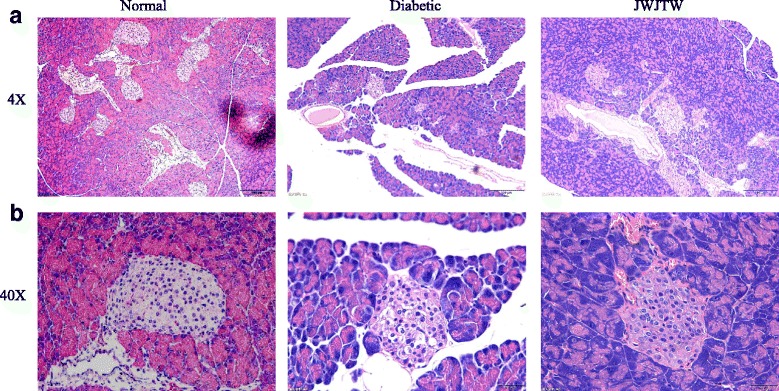
Effects of JWJTW on pancreatic islet number and structure in diabetic rats. **a** Representative hematoxylin and eosin staining of pancreatic islets, 4×. **b** Representative hematoxylin and eosin staining of pancreatic islets, 40 ×

The hematoxylin-eosin (HE) staining of pancreas tissue demonstrated that STZ injection and high-sucrose-high-fat diet administration elicited severe injury to the pancreas. For example, the islet cell number decreased, and the diameter of pancreatic islets was diminished (Fig. 6a). Additionally, the structure of the pancreatic islets was disordered, vacuoles appeared, and nuclei were swollen (Fig. 6b). JWJTW administration produced a moderate expansion of islets, and pancreatic injury was significantly reduced.

### Effects of JWJTW on insulin and PDX-1 protein expression in pancreas of rats

**Fig. 7:**
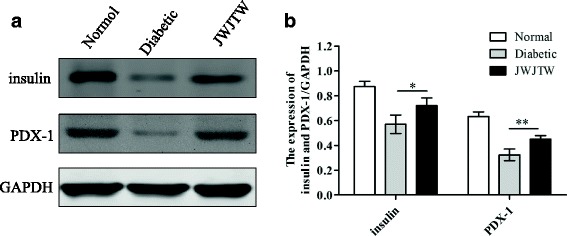
Effects of JWJTW on insulin and PDX-1 protein expression in pancreatic islets of rats. **a** Western blot analysis of insulin and PDX-1 expression in pancreatic islets. **b** Bar graph of the signals for insulin and PDX-1. ^*^ *p* < 0.05, ^**^ *p <* 0.01 (*n* = 5)

The Western blot analysis revealed that the expression of both PDX-1 and insulin in the pancreatic tissues was significantly decreased in diabetic rats compared with the normal group, while diabetic rats treated with JWJTW exhibited a greatly increased expression of PDX-1 and insulin (Fig. 7a, b ^*^ *p* < 0.05, ^**^ *p* < 0.01).

### Effects of JWJTW on the insulin signaling pathway in skeletal muscle of diabetic rats

**Fig. 8:**
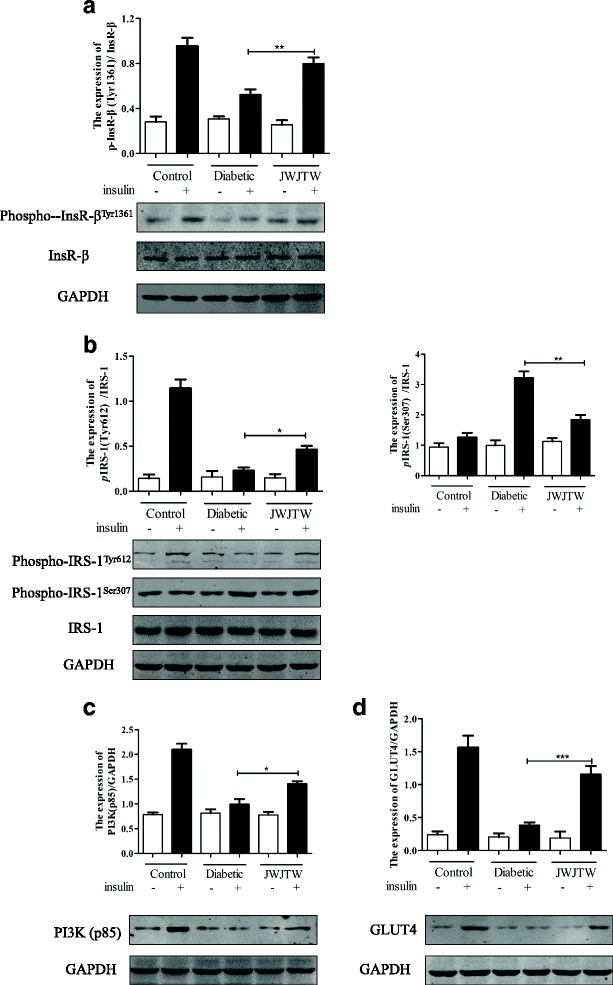
Effect of JWJTW on insulin signaling in skeletal muscle of diabetic rats. Western blot analysis of phospho-InsR-β^Tyr1361^(**a**), phospho-IRS-1^Tyr612^ and phospho-IRS-1^Ser307^ (**b**), PI3K (p85) (**c**) and GLUT4 (**d**). ^*^ *p* < 0.05, ^**^ *p <* 0.01, ^***^ *p* < 0.001 (*n* = 5)

As shown in Fig. 8, there was no significant difference in insulin signaling pathway-related protein expression among the three groups without insulin stimulation; these proteins were all expressed at a low level. With insulin stimulation the degree of IRβ tyrosine phosphorylation (Fig. 8a, ^**^ *p* < 0.01) in the skeletal muscle of the diabetic group rats was decreased significantly compared with that of the normal group rats but the 100-day treatment with JWJTW reversed this downward trend. The decrease in IRS-1 tyrosine phosphorylation levels in the muscle of diabetic rats was also ameliorated by JWJTW treatment, and IRS-1 serine (Ser307) phosphorylation in the same group was markedly downregulated with JWJTW treatment (Fig. 8b, ^*^ *p* < 0.05, ^**^ *p <* 0.01). Once phosphorylated IRS is activated, it combines with the PI3K regulatory subunit p85 and activates the downstream signaling pathway. After the 100-day JWJTW treatment, PI3K regulatory subunit p85 increased significantly in the skeletal muscles of the diabetic animals (Fig. 8c, ^*^ *p* < 0.05). Moreover, the expression of GLUT4 was increased by JWJTW (Fig. 8d, ^***^ *p* < 0.001). Thus, the impaired insulin signaling pathway could be partly restored by JWJTW.

## Discussion

Jiao-Tai-Wan is a traditional Chinese herbal formula that has been demonstrated to have antidiabetic effects in many animal experiments [20–22]. To address the complexity of clinical symptoms in diabetic patients, *Astragalus membranaceus*, *Herba Gynostemmatis*, *Radix Puerariae Lobatae*, *Folium Mori* and *Semen Trigonellae* were added to the Jiao-Tai-Wan prescription to yield JWJTW. Although JWJTW has achieved great antidiabetic effects in clinical applications and animal models, the specific mechanism is not clear. From the animal experiments in this study, we demonstrated that the prescription JWJTW consisting of the above mentioned drugs could ameliorate type 2 diabetes by improving β cell function and reducing IR in rats.

Diabetic patients have impairments in glucose tolerance, insulin release function and insulin tolerance. This study found that JWJTW could ameliorate this abnormal condition in diabetic rats. It is now clear that IR is not solely a disorder of carbohydrate metabolism but also involves alterations in lipid metabolism [23]. It is well known that uncontrolled T2DM is accompanied by increased serum lipid levels. An encouraging result of this research was that 100-day treatment with JWJTW reduced the elevated levels of TC, TG, LDL and FFA in diabetic rats.

The disorder of glucose metabolism in diabetes is partially attributed to oxidative stress. Hyperglycemia leads to the over-production of free radicals, which can exert deleterious effects on the function of β cells, rendering them susceptible to oxidative stress [24, 25]. Hyperglycemia can also degrade antioxidant enzyme defenses, thereby allowing reactive oxygen species (ROS) to damage cells and tissues. NO, a type of reactive nitrogen species that can activate oxidative stress, was reduced by JWJTW in this study. Moreover, the mechanism of impairing NO-mediated reactions is related to superoxide anions [26]; superoxide anions are metabolized to hydrogen peroxide by SOD, which is widely distributed throughout the body [27]. GSH, a strong cellular antioxidant present in many metabolic pathways [28], can reduce different oxidants after the addition of its hydrogen atom. In these reactions, two GSH molecules transform into one molecule of oxidized glutathione (GSSG). This reaction catalyzes the enzyme GSH-px [29] in cells. Our research also revealed that SOD and GSH-px activity in pancreas homogenates were both increased by JWJTW treatment. JWJTW also decreased the lipid peroxidation product, MDA in pancreas homogenates. In addition, researchers have revealed that ROS are closely related to the apoptosis of pancreatic β cells [30], as is the morphology of the pancreatic islets [31]. Our research further revealed that JWJTW administration could partly repair the damaged structure of pancreatic islets caused by STZ and a high-sucrose-high-fat diet in diabetic rats.

It has been reported that ROS can not only induce the apoptosis of pancreatic β cells but also decrease the expression of PDX-1 [32]. PDX-1 is regarded as a central transcription factor that regulates pancreatic development and islet β cell function and maintains β cell-specific gene expression [33]. This study demonstrated that the decreased expression of PDX-1 caused by STZ and a high-sucrose-high-fat diet was upregulated by JWJTW treatment. It has been demonstrated that PDX-1 can regulate the expression of a lot of genes which are involved in maintaining beta-cell identity and function, such as insulin, glucokinase and inslet amyloid polypeptide [34]. In this research, JWJTW also improved insulin expression in the pancreas of diabetic rats by increasing PDX-1 expression.

IR is a general metabolic disorder that is attributable to the inefficient function of insulin in skeletal muscle, liver and/or adipose tissue. It has been documented that the effects of insulin on glucose uptake and metabolism are much greater in skeletal muscle than in liver and adipose tissue [35]. Defects in the insulin signaling cascade leading to impaired glucose utilization are believed to play a key role in the pathogenesis of IR [36]. Furthermore, studies have provided direct evidence of a complete biochemical pathway involving the insulin receptor, IRS-1, and PI3K that can account for important physiological actions by which insulin stimulates glucose uptake in muscle [37]. The present study demonstrated that STZ injection and a high-sucrose-high-fat diet caused reductions in the insulin receptor levels and the efficiency of its tyrosine phosphorylation in the muscle of rats. Our findings showed that JWJTW increased the level of IRβ tyrosine phosphorylation in the muscle of diabetic rats and suggest a beneficial role of JWJTW in the insulin signaling pathway.

Once activated, InsR binds to IRS-1 and activates its phosphorylation. Serine phosphorylation of IRS-1 has been proposed as a general mechanism for the functional inhibition of the IRS-1 protein, and Ser307 phosphorylation is recognized as a molecular indicator of IR [38]. Tyrosine phosphorylation of IRS-1 leads to the binding of PI3K [38] and the activation of its enzymatic activity, a necessary step for the translocation of GLUT4 to the plasma membrane, which consequently elevates the rate of cellular glucose uptake in response to insulin [39].Our results showed that JWJTW treatment significantly inhibited the upregulation of phospho-IRS-1(Ser307) and the downregulation of IRS-1 tyrosine phosphorylation in the muscle of diabetic rats induced by STZ and a high-sucrose-high-fat diet. PI3K, which is downstream of IRS, plays a pivotal role in the insulin signaling pathway and was also increased by JWJTW. Our data also showed that JWJTW treatment achieved a definite improvement in the decreased GLUT4 expression in the skeletal muscle of diabetic rats.

## Conclusions

This study confirmed that JWJTW can ameliorate type 2 diabetes by improving β cell function and reducing IR in diabetic rats. Our results provide a further reference for the elucidation of the specific mechanisms underlying the hypoglycemic effect of JWJTW, and they also provide evidence for the clinical use of JWJTW to treat type 2 diabetes.

## Abbreviations

BSA: Bovine serum albumin
FBG: Fasting blood glucose
FFA: Free fatty acids
GLUT4: Glucose transporter 4
GSH-px: Glutathione peroxidase
HE: Hematoxylin-eosin
HPLC: High-performance liquid chromatography
HUST: Huazhong University of Science & Technology
ig: Intragastric Administration
IGT: Impaired glucose tolerance
InsR: Insulin receptor
IR: Insulin resistance
IRS: Insulin receptor substrate
IRT: Insulin release test
ITT: Insulin tolerance test
JWJTW: Jia-Wei-Jiao-Tai-Wan
LDL: Low-density lipoprotein
MDA: Malondialdehyde
NAFLD: Nonalcoholic fatty liver disease
NO: Nitric oxide
OGTT: Oral glucose tolerance test
PDX-1: Pancreatic and duodenal homeobox 1
PI3K: Phosphatidylino-sitol3-kinase
pInsR: Phospho-insulin receptor
pIRS: Phospho-insulin receptor substrate
PMSF: Phenylmethanesulfonyl fluoride
ROS: Reactive oxygen species
SOD: Superoxide dismutase
STZ: Streptozotocin
T2DM: Type 2 diabetes mellitus
TBST: Tris-buffered-saline and Tween 20
TC: Total cholesterol
TG: Triglyceride
